# Secondary school teachers’ experiences related to learner teenage pregnancies and unexpected deliveries at school

**DOI:** 10.4102/hsag.v24i0.1079

**Published:** 2019-02-25

**Authors:** Antoinette du Preez, A. Johan Botha, Tinda Rabie, Dudu G. Manyathi

**Affiliations:** 1 NuMIQ Research Focus Area, North-West University, South Africa; 2 Edu-HRight Research Unit, Faculty of Education, North-West University, South Africa

## Abstract

**Background:**

The incidence of learner teenage pregnancies can be reduced, provided that the major stakeholders, which include the Department of Education and the Department of Health, combine to address this issue. Despite the implementation of Life Orientation as a school subject, which focuses on sexual behaviour, health, decision-making, pregnancy risk, sexually transmitted infections and HIV and AIDS, the prevalence of learner teenage pregnancies at secondary schools remains alarmingly high.

**Aim:**

The purpose of this study was to explore and describe teachers’ experiences of learner teenage pregnancies in secondary schools in a province in South Africa.

**Setting:**

Secondary schools in KwaZulu-Natal.

**Method:**

A qualitative approach with exploratory, descriptive and contextual strategies was used in this research. Semi-structured interviews were conducted with teachers who had been selected through purposive sampling. Tesch’s steps of systematic open coding were used to analyse all of the interviews.

**Results:**

The results that emerged during the data analysis indicate that firstly, teachers’ experiences of having pregnant learners in their classrooms are negative as these learners are frequently absent from school, perform badly and drop out of school. Secondly, teachers’ experiences related to unexpected deliveries are coloured by their lack of the necessary skills and competencies to deal and their resultant insecurity. Thirdly, the teachers feel that they do not receive the assistance they need to deal with teenage pregnancies as well as unexpected deliveries.

**Conclusions:**

Collaboration between and continuous support from the Department of Health and the Department of Education are crucial if teenage pregnancies at secondary schools are to be handled with greater success. Support from health care personnel should include guidance on contraception and health campaigns should target both the teachers and secondary school learners. Furthermore, coping strategies for the teachers should form part of the preservice curriculum of teachers.

## Introduction and background information

The role that teachers can play in reducing the incidence of teenage pregnancies among learners has been debated worldwide. Statistics from Europe, the United States of America (USA), sub-Saharan Africa and South Africa suggest that various interventions aimed at reducing teenage pregnancies have had little or no success (Chaibva, Ehlers & Roos 2009).

South Africa is experiencing an increase in teenage pregnancies, particularly among teenagers aged between 15 and 18 who are at secondary schools (Sibeko 2012). In South Africa, just over 31.5% of teenage girls give birth by the age of 18 (Karra & Lee 2012). KwaZulu-Natal (KZN) province has a high incidence of teenage pregnancies among schoolgirls (Panday et al. 2009). KwaZulu-Natal is one of the top three provinces regarding teenage pregnancies (South African Demographic and Health Survey [SADHS] 2016). On 14 June 2012, the Member of the Executive Council for education in KZN said that thousands of schoolgirls had become pregnant in KZN and that the province was facing a serious predicament (Sibeko 2012). The rising incidence of teenage pregnancies poses social, emotional and physical health risks to teenagers (Ncube 2009). These socio-economic, health and educational challenges highlight the need for managerial strategies (Panday et al. 2009).

Maholo, Maja and Wright (2009) conducted research on teenage pregnancies that revealed that high maternal mortality rates and infant mortality rates are associated with an increased incidence of sexually transmitted infections. The aim of their research was to address the Millennium Development Goals 4, 5 and 6 through the implementation of measures to reduce the incidence of teenage pregnancies (Maholo et al. 2009; World Health Organization [WHO] 2011). They concluded that support in secondary schools was necessary not only for pregnant teenagers but also for teachers who lack the skills to assist in teenage deliveries during school hours and are traumatised by having to do so.

Teenage pregnancies are a matter of great concern to health professionals as early pregnancy may lead to serious, even life-threatening, conditions in these young mothers. Pregnancy-induced hypertension, diabetes, placenta abruptio and premature births are more common among teenagers than among adult women (James, Van Rooyen & Strumpher 2010).

In South Africa, the Life Orientation Curriculum Assessment Policy Statements (CAPS) (South Africa 2011a) are concerned with facilitating the holistic development of learners. This includes creating opportunities for secondary learners to acquire the knowledge and awareness of sexual behaviours that could lead to pregnancy and the associated health risks (Panday et al. 2009). The goal of Life Orientation teachers should be to offer guidance to learners related to their personal, social and physical development (South Africa 2003). The implementation of Life Orientation (South Africa 2011a) programmes has supported teachers’ endeavours. These programmes focus on life skills as a means of addressing the negative impact of teenage pregnancies (Bastable & Dart 2007). In order to be successful, Life Orientation teachers require the support of health professionals and other health stakeholders (South Africa 2011a). Teachers and nurses should work together to set goals, solve problems and make decisions that would help teenagers to make informed choices (Cornerstone Consulting Group 2001).

Youth development programmes that include sex education (Manlove et al. 2009) are essential for delaying first sexual encounters and decreasing the rate of teenage pregnancy so the South African Department of Health (DOH) recommends a universal approach to school-based sex education (Panday et al. 2009). In the interest of the learners’ overall well-being, schools are expected to emphasise abstinence and safe sex to prevent sexually transmitted infections and unwanted pregnancies (South Africa 2010). Another concern should be that teenagers are at risk of giving birth to grossly abnormal babies. Marieb and Hoehn (2007) reported that teenagers tend to do so more frequently than adult women. One of the factors involved is that teenagers are married to blood relatives from the age of 13. These teenage girls are under particular pressure to produce an heir; they could be ostracised by their communities should they fail to do so (Lehana & Van Rhyn 2003).

The *South African Schools Act* 84 of 1996 (South Africa 1996) aims to protect learners. Consequently, it promotes abstinence among schoolgoers. However, this does not imply that the educational environment may discriminate against any learner who falls pregnant or fail to respect her right to confidentiality (Bhana et al. 2010). Education White Paper 6 (South Africa 2001), which addresses lifelong learning through education and training, emphasises that all children and adolescents require support, respect and acceptance. Differences among teenage learners should be respected, whether they be age, gender, ethnicity, language, class, disability, HIV status or teenage pregnancy (South Africa2011b). This is supported by the *South African Schools Act* 84 of 1996 (South Africa 1996), which states that all learners should be treated with respect and fairness.

Teenage pregnancy has a negative impact on the pregnant learners themselves. Their situation leads to exclusion from peer groups, poor performance or even to their dropping out of school. However, sometimes their pregnancy is the result of a deliberate act because it is seen as fashionable. According to Mpofu (2012), pregnancy also occurs because some males no longer feel culturally obligated to protect women from unwanted pregnancies. Some males refuse to use condoms. By not doing so, they not only expose teenage girls to unwanted pregnancies but also to sexually transmitted infections (Willan 2013). Masweneng (2018) found that two other factors played a role in the high incidence of teenage pregnancies and early sexual behaviour: the lack of parental guidance as well as substance abuse. Yet another possible reason for learners’ falling pregnant is to access child support grants (Squires 2011). For Savides (2018), what is needed to redress the situation is a clear policy.

During 2013, a total of 99 000 secondary learners became pregnant at a rate of 271 per day (Pillay et al. 2018). In KZN province in South Africa, 21 000 teenage pregnancies were recorded among girls younger than 18 between April 2012 and March 2013 (Peters 2014). Statistics recorded in 2014 show that there had been 343 pregnancies during the previous 4 years at the four secondary schools in KZN used in this study: 100 in 2010, 85 in 2011, 95 in 2012 and 63 in 2013.

### Research problem statement

Secondary schools find teenage pregnancies problematic because they lack the necessary management strategies and support services to assist the learners and teachers (Chigana & Chetty 2008). Factors that contribute to teenage pregnancies are that secondary school learners make little or no use of the contraceptives that are freely available at all state primary health care facilities (South Africa 2012). Teachers and nurses might also not be adequately mentoring and supporting teenage girls at secondary schools (Ncube 2009). Mantell et al. (2006) suggest that teachers teaching the Life Orientation curriculum might lack the particular knowledge required to teach about sensitive matters such as sexual behaviour, sexual health, decision-making regarding sexuality, risk of pregnancy, sexuality and transmitted infections, including HIV and AIDS preventive measures. Parents also tend to be outspoken in their criticism of Life Orientation teachers who teach about sexuality and/or sexual behaviour, thus hindering their work (Panday et al. 2009). Maholo et al. (2009), MacLeod and Tracey (2010), as well as Mantell et al. (2006), raise quite another possible reason: social norms and cultural values might influence early sexual behaviour.

According to the *South African Schools Act* 84 of 1996 (South Africa 1996), teenagers should remain at school even if pregnant (Ramulumo & Pitsoe 2013). They are entitled to make use of antenatal services, which could interfere with regular school attendance and teaching–learning (Chen et al. 2006). Teachers find themselves having to deal not only with teenage pregnancies but also with a number of interrelated issues that complicate the teaching–learning situation. Not only do they lack the necessary experience, knowledge and skills to handle teenage pregnancies but also those to deliver babies.

### Purpose and objectives

The purpose of the study was to explore and describe teachers’ experiences related to teenage pregnancies in secondary schools in the KZN province of South Africa. The objectives of the study were to explore and describe: (1) secondary school teachers’ experiences of teenage pregnancies at school, (2) their experiences of unexpected deliveries at school, (3) how they cope with unexpected deliveries at school and also to (4) provide recommendations on what is needed to enable secondary school teachers to cope with teenage pregnancies and manage unexpected deliveries at school.

### Definitions of key concepts

*Secondary school* is an educational institution that provides for the educational needs of learners during the final stage of schooling, also known as the Further Education and Training Phase (FET). All secondary schools have to be registered by the Department of Education (DOE) as a private or public institution and have to provide a safe and conducive environment for teaching and learning (Myburgh & Poggenpoel 2003; South Africa 1996).

*Teacher* refers to a person who has received formal training and teaches, educates or trains other persons. This person provides professional educational services including professional therapy at any educational institution and his or her appointment is subject to the terms and conditions of the employment rules and regulations of the *South African Schools Act* 84 of 1996 (South Africa 1996).

*Learner* refers to any person who is obliged to receive education in terms of the *South African Schools Act* 84 of 1996 (South Africa 1996). In this article, the term *learners* refers to teenage learners who fall pregnant while still attending secondary school in KZN province and who may unexpectedly give birth while at school. Thus, teenage pregnancies in this context refer to pregnant learners attending secondary schools in KZN province.

*Life Orientation* is a compulsory school subject prescribed by the Department of Basic Education, stated in the CAPS and presented at secondary schools in the Senior Phase and FET phase (South Africa 2011a).

*Teenager* refers to an adolescent between the ages of 10 and 18, who has not yet reached legal adulthood (Mosby’s Dictionary 2006). In this study *secondary school learners* refers to learners between 13 and 18 years of age.

*Teenage pregnancy* is the term used by the World Health Organization (2011) to refer to an adolescent between the age of 13 and 18 years old who is pregnant. In this article, the pregnant teenager concerned is still attending secondary school (Kanku & Mash 2010).

*Unexpected delivery* is used to refer to a delivery that does not occur in an obstetric unit. Thus, a delivery at school is regarded as an unexpected delivery because the birth of the child or the period of parturition does not take place in the delivery unit at a hospital (Mosby’s Dictionary 2006:525).

## Materials and methods

### Design

This qualitative research approach was exploratory, descriptive and contextual. This design made provision for obtaining sufficient and relevant information from the participants (Burns & Grove 2011).

### Population

The population included both male and female teachers at four secondary schools in a subdistrict of KZN province in South Africa. All the teachers had diplomas or degrees and had at least 5 years’ teaching experience. Table 1 presents an interpretation of the population of the four schools in the selected district.

**TABLE 1:** Secondary school demographic profile.

Secondary Schools	School A	School B	School C	School D
Teachers	42	17	23	28
Schoolgirls	300	250	257	220
Number of pregnancies in 2010	30	40	12	18
Number of pregnancies in 2011	25	43	5	12
Number of pregnancies in 2012	27	57	5	6
Number of pregnancies in 2013	50	5	3	5

### Sampling and participants

Purposive sampling was employed to select teachers at secondary schools with specific experience within a specific education district in KZN province (Maree & Pietersen 2016:198). The sample consisted of 19 teachers employed at secondary schools who were willing to participate. Selection criteria required participants to have a teaching qualification, to speak English, to have had at least 5 years’ teaching experience and to have experience of dealing with teenage pregnancies at their respective schools.

### Demographic profile of the participants

Of the 19 teachers, 47.0% (*n* = 9) had 5–10 years’ teaching experience, while 32.0% (*n* = 6) had 11–16 years’ teaching experience and 21.0% (*n* = 4) had 17–25 years’ teaching experience.

### Data collection procedures

Data were collected during 2014 by using semi-structured individual interviews (Brink, Van der Walt & Van Rensburg 2012; Nieuwenhuis 2016; Polit & Beck 2012).

During the semi-structured individual interviews, an interview schedule with predetermined questions based on the research topic was used. The initial interview questions were: How do you experience teenage pregnancies at your school? How do you experience unexpected deliveries at your school? How do you cope with teenage pregnancies and unexpected deliveries at your school? These interviews were conducted in English, lasted between 20 and 40 min and were audio recorded with the consent of the participants. This made it possible to make verbatim transcripts of the interviews that could be analysed.

### Data analysis

Tesch’s (1990) steps of systematic open coding were used to analyse the data (Creswell 2014). This data analysis process began with reading and rereading the typed verbatim transcripts of the interviews to gain an understanding of the whole. Any ideas that came to mind were jotted down during reading of all the transcribed interviews. Similar topics that emerged were combined and the resultant topics were abbreviated so they could be used as codes. Codes were applied to appropriate segments and then the most descriptive of each topic were used to compile a list of categories. All related categories were then grouped, making it possible to make final decisions on the themes and subthemes that had emerged during the data analysis (Creswell 2014).

### Ethical considerations

Ethical approval (number NWU-00143-13-S1) was granted by the North-West University’s Ethical Committee. Permission to conduct the research was obtained from the Department of Education in KZN province. All those who participated in this research did so voluntarily. Participants were not harmed emotionally or physically and they were assured that they were free to withdraw from the study at any time, without any consequences. The participants were also informed about the nature of the research, as well as its objectives and method of data collection, prior to the interviews. Only teachers who agreed to participate and signed the voluntary consent form were selected as participants. The interviews did not begin until that point. Great care was taken not to record the participants’ names or any information that could identify them during the transcription of the audio-recorded interviews.

## Results

From the data analysis, three themes emerged: teachers’ overall experience related to teenage learners’ pregnancies, experiences regarding unexpected deliveries at school and teachers’ own recommendations on how to enhance teachers’ coping abilities. Verbatim quotations from the transcripts are provided as examples to illustrate the identified themes.

### Overall experiences of secondary school teachers relating to teenage learners’ pregnancies

Three factors cited by the participants influenced the teachers’ overall perceptions of their experience related to teenage learners’ pregnancies: the effect on the teaching–learning situation in the classroom, absenteeism and poor school performance or dropping out of school. Specific problems related to the teaching–learning situation that the teachers mentioned are as follows: a tendency to fall a sleep in the classroom (Mpanza 2012:56; Sibeko 2012); comments made by other learners; pregnant learners feeling sick; and mood swings that affected their attitude to classroom activities. These had a negative impact not only on the interaction between the secondary school teacher and the pregnant teenager learner but also on other learners.

‘We find that they affect the whole situation in other learners; they also affect other learners.’ (P1, female, teacher)

‘Eh sometimes it is sickness, sometimes there are remarks from the class/learners.’ (P7, female, teacher)

‘Eh somebody sleeping in the class with learners’ remarks, somebody is pregnant.’ (P7, female, teacher)

Factors contributing to absenteeism included appointments at the antenatal clinic and the postnatal clinic and at the immunisation clinic, so the baby could get its shots (Mpanza 2012:3; Sibeko 2012), embarrassment about being pregnant or sickness because of pregnancy, especially during the first trimester (Mpanza 2012; Panday et al. 2009) and the actual birth (Gyan 2013). Another reason for absenteeism is the need to collect social grants (Willan 2013). The social grant policy was a matter of great concern for the teachers, as they felt this grant encouraged early pregnancies: learners saw the meagre grant as a means of contributing to the hard-pressed household (Squires 2011:5).

‘I think the first problem that we have is that we have is that on the first trimester when the, children don’t want the educators to see that they are pregnant. First thing is that they absent themselves at schools, uhm, [*maybe because*] they [*don’t*] want to go or may be because they are still vomiting and all that.’ (P1, female, teacher)

‘Yes, it interferes with the performance at school; the experience is based on performance, experience and absenteeism.’ (P2, female, teacher)

‘… absent for several time, for one I think that maybe they go attend antenatal clinic.’ (P6, female, teacher)

### Experiences regarding the unexpected births of babies at schools

These feelings experienced by the teachers were directly related to their lack of skills and competencies and their insecurity about delivering babies on their own. Sibeko (2012) reported that an increase in teenage pregnancies and unexpected births at school made teachers feel overburdened. Participants in the current study verbalised that they were trained as teachers in the education system, not as midwives, and that there was not even basic equipment, such as gloves, at the school. Their view is supported by Mpanza (2012), who underlined the heavy demands made on teachers to do their job and to be midwives. Participants in another study perceived the policy context to be shifting continuously, changing schools into new sexual environments and new places of freedom for teenagers (Bhana et al. 2010:876).

‘That depends on to saying we are not trained for midwife.’ (P12, female, teacher)

‘Eish, it is difficult because the first thing you are not a doctor not a nurse; you are not a trained as a person who can deliver the baby.’ (P3, female, teacher)

‘We don’t have gloves – no provision for such situation, no precautions for such a situation; it is worse for performing skills such as deliveries.’ (P2, female, teacher)

Participants indicated they felt insecure about handling births. Though there are policies in place regarding learners’ teenage pregnancies, they do not address the issue of handling of emergency deliveries at secondary schools.

‘… there is an eminent delivery happening in the school, now I have to take the learner with my car to the hospital; this is a very high risk encounter, and my car was dirty because of the delivery in my car.’ (P4, female, teacher)

‘They don’t watch such a learner because, eh, we never trained as midwives, so it is not easy to touch such a learner; so maybe it can do maybe is to call for help, so as I am afraid of blood maybe it is just for diseases – just I am afraid of blood and my blood just boils when I see the blood.’ (P12, female, teacher)

Participants reported a lack of assistance from staff members and from Emergency Medical Rescue Services (EMRS) in cases of emergencies and unexpected births at secondary schools. Fokazi (2013) mentioned that delays could occur as a result of poor road infrastructure and the limited availability of medical services. According to the DOH (South Africa 2011b), there is a severe shortage of ambulances, especially in KZN. Many schools are located in rural areas with poor infrastructure. Some participants in the current study mentioned that it would be quicker to use their own vehicles to rush a teenager to hospital than to wait for EMRS. Participants felt insecure about rendering assistance to these teenagers and their babies because should anything go wrong, the teacher could be charged with negligence (Mpanza 2012).

‘Uhm, not I, but I had that some other cases and in this immediately they just call the ambulance and they take the learner to the sick bay here so that she will relax.’ (P10, female, teacher)

‘It was about, it was about to go, yes, we had to rush; calling the ambulance, you see, no ambulances came during that time. We have to use, I had [*to*] use my own car, taking the child to the hospital, because there was some water coming out, you see, and we had to rush, and it was really a risk to me. It used to happen and in other schools it really happened in the classroom.’ (P3, female, teacher)

### Recommendations to enhance the skills teachers need to cope with births at school

Five main issues emerged under the theme of recommended ways of enhancing teachers’ capacity to cope with teenagers giving birth at school. These included recommendations to the DOH, DOE, school governing body (SGB), collaboration between the DOH and DOE and regular awareness campaigns.

In their view, the DOH could greatly assist secondary school teachers if they appointed health professionals to be based at schools or who regularly visit secondary schools to assist pregnant learners. The participants also stated that health professionals should offer contraceptive services, antenatal care and health education targeted at teenage learners.

‘… maybe it will be easy for the government to appoint any health worker to be within the premises at school to cater for those who are in that situation at that time, because I don’t think it is easy for us as teachers to leave unattended learners and attend those learners who is in labour.’ (P4, female, teacher)

Some participants suggested that the DOH should provide further support. Some years ago, the DOE compiled and implemented the *South African Schools Act* 84 of 1996 (South Africa 1996), addressing the need for teenage pregnancy policies. This policy emphasises learners’ right to freedom of choice, promotes respect for an individual’s dignity and encourages educators to promote abstinence or delayed initiation of sexual activities (Sibeko 2012; South Africa 1996; Wilan 2013). This is in accordance with the Integrated School Health Policy (ISHP), which argues for the need for school health care specialists to address the health of schoolgoers (South Africa 2012). The participants felt that some of the rights accorded to learners and the help offered to mothers in the form of grants were problematic.

‘The government can do something if only it can revise the rights, because it is these rights and these grants which are encouraging the kids to get pregnant and not to behave.’ (P2, female, teacher)

Participants mentioned that the SGB could also assist by developing a policy that made parents responsible for monitoring pregnant learners. The SGB, on which parents are represented, seeks to promote the best interests of the community (Mpanza 2012). It is not only the responsibility of the secondary school teachers but also that of the parents and learners in the school.

‘… some parents, they take the learner home, and some say let’s monitor the learner until just before they deliver.’ (P10, female, teacher)

The participants also recommended collaboration between the DOH and DOE to realise some of the goals envisaged in the ISHP (South Africa 2012), such as having health care professionals who specialise in school health services. This is a major component of the Care and Support for Teaching and Learning programme (South Africa). The DOH should provide services aimed at the prevention of teenage pregnancies and any related emergencies within the school environment. This should help to optimise the available resources and create an enabling environment for teachers to assist the learners (Bhana et al. 2010; Panday et al. 2009). The stakeholders should promote teenage sexual health and regularly update the knowledge presented to learners.

‘The support from the Department of Education as well as from the Department of Health.’ (P8, female, teacher)

Participants emphasised the importance of having awareness campaigns that offered sex education designed to prevent teenage pregnancies and involved parents.

‘The campaigns that involve both, uhm, health and education and is also thinking that the government should see to it that, uhm, we, that the government provides public schools with, uhm, a professional nurse for such cases.’ (P7, female, teacher)

‘I think if [*the*] Department of Education can, what you called, they can workshop more learners about, eh, teenage pregnancy, they must be aware of everything why we fall and all that and according and Department of Health.’ (P10, female, teacher)

### Limitations

The study was conducted in only one district in KZN province and was limited to secondary school teachers’ experience. There were no learner participants. The results merely serve to offer broad indications of what might be true for teachers of the greater school population.

### Recommendations for future research

Collaboration between the DOE and the DOH should support teachers to understand the importance of contemporary approaches when implementing the Life Orientation curriculum in schools. In turn, the DOH should provide relevant health facilities with highly skilled health personnel who can operate effectively in school environments. These health facilities should engage in regular health campaigns at schools that include contraceptive services, rather than being limited to them. Future research should focus on learners’ experiences with regard to support received from schools during and after teenage pregnancies. Learners’ perceptions of how effective the Life Orientation curriculum is in preventing teenage pregnancies should also be investigated.

## Conclusions

The participants in this study were concerned about the negative effects of teenage pregnancies at secondary schools, particularly on teachers and learners and the teaching–learning process. What emerged clearly is that teachers would appreciate regular visits to the schools by health care professionals to provide antenatal care and support to pregnant learners, to assist during births at schools and to offer contraceptive guidance and services to learners. The health care professionals should offer a great deal more support to secondary school teachers, particularly with regard to teenage learner pregnancy and unexpected deliveries at rural schools.

## References

[CIT0001] Bastable S.B. & Dart M.A., 2007, ‘Developmental stages of the learner’, in Bastable S.B. & Dart M.A. (eds.), *Nurse as educator: Principles of teaching and learning practice*, pp. 147–198, 3rd edn., Sudbury, MA, Jones & Bartlett Publishers.

[CIT0002] Bhana D., Morrell R., Shefer T. & Ngabaza S., 2010, ‘South African teachers’ responses to teenage pregnancy and teenage mothers in schools’, *Cultural, Health and Sexuality* 12(8), 871–883. 10.1080/13691058.2010.500398 20665296

[CIT0003] Brink H.I., Van der Walt S.J.L. & Van Rensburg G., 2012, *Fundamentals of research methodology for healthcare professionals*, 3rd edn., Juta, Cape Town.

[CIT0004] Burns N. & Grove S.K., 2011, *Understanding nursing research*, 7th edn., W.B. Saunders, Philadelphia, PA.

[CIT0005] Chaibva N.C., Ehlers V.J. & Roos J.H., 2009, ‘Midwives’ perceptions about adolescence utilization of public prenatal services in Bulawayo, Zimbabwe’, *Midwifery* 26(6), e16–e20. 10.1016/j.midw.2009.01.001 19246134

[CIT0006] Chen X.K., Wen S.W., Fleming N., Demissie K., Rhoads G.G. & Walker M., 2006, ‘Teenage pregnancy and adverse birth outcomes: A large population based retrospective cohort study’, *International Journal of Epidemiology* 36(2), 368–373. 10.1093/ije/dyl284 17213208

[CIT0007] Chigana A. & Chetty R., 2008, ‘Teen mothers and schooling: Lacunae and challenges’, *South African Journal of Education* 28(2), 261–281.

[CIT0008] Cornerstone Consulting Group, 2001, *Program approaches in teen pregnancy prevention: Best practices and effective and promising programs*, viewed 29 September 2014, from http://www.pbchd.com/pdfs/Best_Practices_PBC.pdf

[CIT0009] Creswell J.W., 2014, *Research design: Qualitative, quantitative and mixed methods approach*, 4th edn., Sage, California, CA.

[CIT0010] Fokazi S., 2013, *Melomed-hospital-Mitchells-Plain vs. Cape Argus*, viewed 10 July 2013, from http://www. presscouncil.org.za/melomed-hospital-mitchells-plain-vs-cape-argus-2471

[CIT0011] Gyan C., 2013, ‘The effects of teenage pregnancy on educational attainment of girls at Chorkor suburb of Accra’, *Journal of Educational and Social Research* 3(3), 53–60. 10.5901/jesr.2013.v4n3p53

[CIT0012] James S., Van Rooyen D. & Strumpher J., 2010, ‘Model of facilitation of intergenerational reconciliation in teenage pregnancy: A Xhosa perspective’, *African Journal of Nursing and Midwifery* 12(2), 3–13.

[CIT0013] Kanku T. & Mash R., 2010, ‘Attitudes, perceptions and understanding amongst teenagers regarding teenage pregnancy, sexuality and contraception in Taung’, *South African Family Practice* 52(6), 563–572. 10.1080/20786204.2010.10874048

[CIT0014] Karra M. & Lee M., 2012, *Human capital consequences of teenage childbearing in South Africa*, Population Reference Bureau, Washington, DC.

[CIT0015] Lehana T.V. & Van Rhyn L., 2003, ‘A phenomenological investigation of experiences of pregnancy by unmarried adolescents in Maseru: Research’, *Health SA Gesondheid* 8(1), 26–38. 10.4102/hsag.v8i1.113

[CIT0016] MacLeod C.I. & Tracey T., 2010, ‘A decade later: Follow-up review of South African research on the consequences of and contributory factors in teenage pregnancy’, *South African Journal of Psychology* 40(1), 18–31. 10.1177/008124631004000103

[CIT0017] Maholo R.B., Maja T.M.M. & Wright S.C.D., 2009, ‘Relationships, perceptions and the sociocultural environment of teenagers in Soshanguve Secondary School’, *African Journal of Midwifery* 11(2), 48–60.

[CIT0018] Manlove J., Ikramuller B.A., Mincieli L., Holcombe E. & Danish S., 2009, ‘Trends in sexual experience, contraceptive use and teenage child bearing’, *Journal of Adolescent Health* 44(5), 413–423. 10.1016/j.jadohealth.2008.09.006 19380087

[CIT0019] Mantell J.E., Harrison A., Hoffman S., Stein Z.A. & Exner T.M., 2006, ‘The Mpondombili project: Preventing HIV/AIDS and unintended pregnancy among rural South African school-going adolescents’, *Reproductive Health Matters* 14(28), 113–122. 10.1016/S0968-8080(06)28269-7 17101429

[CIT0020] Maree K. & Pietersen J., 2016, ‘Sampling’, in Maree K. (ed.), *First steps in research*, pp. 192–202, 2nd edn., Van Schaik Publishers, Pretoria.

[CIT0021] Marieb E.N. & Hoehn K., 2007, *Human anatomy and physiology*, 7th edn., Pearson Benjamin Cumming, Harlow.

[CIT0022] Masweneng K., 2018, ‘27 learners pregnant at a single school’, *Sunday Times*, 26 July, viewed 29 July 2018, from https://www.timeslive.co.za/news/south-africa/2018-07-26-27-learners-pregnant-at-a-single-school/

[CIT0023] Mosby’s Dictionary of Medicine, 2006, *Nursing and health professionals*, 7th edn., Elsevier, St. Louis, MO.

[CIT0024] Mpanza N.D., 2012, ‘Educators’ experiences in dealing with teenage pregnancy’, PhD thesis, University of Zululand, Empangeni.

[CIT0025] Mpofu M., 2012, *Thousands of teens, children are pregnant*, viewed 12 October 2013, from http://www.iol.co.za/the-star/thousands-of-teens-children-are-pregnant-1.1336665#.VFIxgfmUet8

[CIT0026] Myburgh C.P.H. & Poggenpoel M., 2003, ‘Teacher’s experience of their school environment for health promotion’, *Education* 123(2), 260–267.

[CIT0027] Ncube M., 2009, ‘The knowledge and awareness of grade twelve learners about teenage pregnancy: A case study at Vine College High School’, PhD thesis, University of the Witwatersrand, Johannesburg.

[CIT0028] Nieuwenhuis J., 2016, ‘Qualitative research designs and data-gathering techniques’, in Maree K. (ed.), *First steps in research*, pp. 72–102, 2nd edn., Van Schaik Publishers, Pretoria.

[CIT0029] Panday S., Makiwane M., Ranchod C. & Letsoalo T., 2009, *Teenage pregnancy in South Africa: With a specific focus on school-going learners*, Human Science Research Council, Pretoria.

[CIT0030] Peters S., 2014, ‘Teenagers breeding poverty’, *Daily News*, 23 September, p. 9.

[CIT0031] Pillay S., Sibanda W.,Ghuman M.R. & Coutsoudis A., 2018, ‘Infant feeding practices of teenage mothers attending a well-baby clinic in a public hospital in Umlazi, KwaZulu-Natal, South Africa’, *South African Journal of Clinical Nutrition* 31(1), 14–19.

[CIT0032] Polit D.F. & Beck C.T., 2012, *Nursing research generating and assessing evidence for nursing practice*, 9th edn., Lippincott, Philadelphia, PA.

[CIT0033] Ramulumo M.R. & Pitsoe V.J., 2013, ‘Teenage pregnancy in South African schools: Challenges, trends and policy issues’, *Mediterranean Journal of Social Sciences* 4(13), 755–760.

[CIT0034] Savides M., 2018, ‘Pupil pregnancy: So rife we need a policy on it’, *Sunday Times* 29 July, viewed 29 July 2018, from https://www.timeslive.co.za/news/south-africa/2018-06-29-pupil-pregnancy-so-rife-we-need-a-policy-on-it/

[CIT0035] Sibeko P.G., 2012, ‘The effects of pregnancy on a schoolgirl’s education’, PhD thesis, University of Zululand, Empangeni.

[CIT0036] South Africa, 1996, *The South African Schools Act 84 of 1996*, Government Printers, Pretoria.

[CIT0037] South Africa, Department of Basic Education, 2011a, *Curriculum and assessment policy statement, grade 10-12: Life orientation*, Government Printers, Pretoria.

[CIT0038] South Africa, Department of Basic Education, 2011b, *Strategic plan 2011–2014*, Government Printers, Pretoria.

[CIT0039] South Africa, Department of Basic Education, 2012, *Integrated school health policy*, Government Printers, Pretoria.

[CIT0040] South Africa, Department of Education, 2001, *Education White Paper 6*, Department of Education, Pretoria.

[CIT0041] South Africa, Department of Education, 2003, *National curriculum statement, grades 10-12: Physical sciences*, Department of Education, Pretoria.

[CIT0042] South Africa, Department of Health, 2010, *Saving mothers: Report on the confidential enquiries into maternal deaths in South Africa*, Government Printers, Pretoria.

[CIT0043] South African Demographic and Health Survey (SADHS), 2016, *Key indicators report 2016*, Statistics South Africa, Pretoria.

[CIT0044] Squires L., 2011, *Suffolk country taskforce on teen pregnancies*, viewed 03 October 2014, from http://legis.suffolkcountyny.gov/clerk/cmeet/tptf/2011/tptf_report_051011.pdf

[CIT0045] Tesch R., 1990, *Qualitative research: Analysis types and software tools*, Falmer, New York.

[CIT0046] Willan S., 2013, *A review of teenage pregnancy in South Africa: Experience schooling and knowledge and access to social and reproductive health services*, viewed 03 October 2014, from http://www.rmchsa.org/wp-content/uploads/2013/08/Teenage-Pregnancy-in-South-Africa-2013.pdf

[CIT0047] World Health Organization (WHO), 2011, *Early marriages, adolescence and young pregnancies*, viewed 29 September 2014, from http://apps.who.int/iris/bitstream/10665/23744/1/B130_12-en.pdf?ua=1

